# Longitudinal torsional vibrations of the chain drive system of mine scraper conveyor

**DOI:** 10.1038/s41598-023-36357-0

**Published:** 2023-06-06

**Authors:** Jinnan Lu, Runkun Yang, Jun Mao, Chunxue Xie

**Affiliations:** 1 School of Mechanical Engineering, Liaoning Technical University, Fuxin, 123000 Liaoning China; 2 Fuxin University of Technology Artificial Intelligence and Equipment Industry Technology Research Institute, Fuxin, 123000 Liaoning China

**Keywords:** Mechanical engineering, Electrical and electronic engineering

## Abstract

To deeply analyse the dynamic characteristics of the scraper conveyor during operation, the mechanical characteristics of the coupled longitudinal and torsional vibrational modes under excitation by cargo loading are studied. Based on the Kelvin‒Voigt model and the point-by-point tension method, a model of the coupled longitudinal and torsional vibrations of the scraper chain drive system is established. Then the functional program is constructed and the numerical simulation is carried out. Finally, the correctness of the model is verified by comparison with experiments. The research results reveal the torsional vibration characteristics of the scraper chain drive system under two different working conditions, light load and medium load, and determine the influence area of the torsional vibration of the scraper. The results of this analysis provide a theoretical basis for the subsequent optimization of the scraper parameters, the prediction of scraper chain drive system failure, and the calculation to give an early warning before failure occurs.

## Introduction

Because of its structural characteristics, the scraper conveyor can be regarded as a continuous rigid and flexible coupled structure. The operation of the equipment is often accompanied by corresponding longitudinal and torsion pendulum vibrations. Due to the harsh working environment and the impact of shear large coal rock^1–3^, the chain becomes stuck by the impact load and stops working or even breaks in serious cases^4–6^.

At present, some scholars have performed research on the dynamic characteristics of rigid and flexible coupling of scraper conveyors. Dolipsk et al.^7^ established a dynamic model of the nonuniform load state of a scraper conveyor and conducted computer simulations and an analysis on the working load of a long-distance conveyor. Shuhuan et al.^8^ studied the influence of the change in the load on the dynamic characteristics of the scraper conveyor. Nie et al.^9^ used multiple space-fixed finite elements to simulate the chain drive system, included the distribution and motion form of the cargo load, and solved the dynamic characteristics of each element by the Euler method. Zhang et al.^10^ analysed the change in conveyor chain tension by using an ADAMS simulation, numerical simulation and a state observer. Lianhang et al.^11^ established a mechanical model of the lateral bending of a section of a scraper conveyor and calculated the parameters of the horizontal bending section of the central trough and their relationship. Jun^12^ studied the dynamic behaviour of the longitudinal fluctuation of the scraper conveyor and established a dynamic finite element model. Xiufang^13^ derived the vibration equation and an analytical formula for scraper conveyors under different driving and transportation modes and solved the vibration equation by discretization. Based on the mechanism of the resistance of a chain running under extreme conditions, Li et al.^14^ derived a formula for the resistance of the running chain. Zhang et al.^15^ introduced a method to estimate the tension distribution of the scraper chain drive system, established a mathematical model of the ring chain drive system, and verified the performance of the dynamic model with the model solved by the MATLAB function. Dongsheng et al.^16^ carried out simulation and experimental research on dynamic characteristics of starting and braking of scraper conveyor. Yao^17^ analysed the dynamic characteristics and intelligent control method of drive system of heavy scraper conveyor. Wei^18^ analyzed the harm of the chain tension change of the scraper conveyor, and put forward a monitoring method.

In the analysis of dynamic characteristics under fault conditions, Miao et al.^19^ established a general equation for the longitudinal fluctuation of the chain, determined the boundary and initial value conditions, and analytically solved the mathematical model. MATLAB software was used to simulate the dynamic problems of the scraper conveyor under direct starting and chain failure conditions. Jiang et al.^20^ studied the dynamic characteristics of a scraper conveyor by measuring the vibration signals of the output shaft of the scraper conveyor reducer for different chain speeds, terrains and load conditions. Dongsheng et al.^21^ used numerical simulations to study the meshing transmission of the sprocket and chain of a scraper conveyor. They also analysed the vibration characteristics of the polygon effect of the scraper conveyor chain under two working conditions, with and without a cargo load.

In summary, scholars at home and abroad have achieved fruitful results in the study of the dynamic characteristics of scraper conveyors. However, from the perspective of research content, only a few chain links are considered in dynamics simulation by ADAMS software, thus the tension fluctuation state of the whole machine cannot be reflected well. The numerical simulation did not consider a series of torsional vibrations caused by the uneven force of the double chain. Thus, the simulation could not fully reflect the dynamic characteristics of the whole machine under multiple working conditions and impact loads.

In view of the limitations of prior research, this study applies the Kelvin‒Voigt model and point-by-point tension method to establish a coupling model of the longitudinal and torsional vibrations of a scraper conveyor. This research considers the uneven force of double chains under excitation conditions such as sudden load change, and the results reveal the mechanical characteristics of longitudinal and torsional vibrations of the scraper chain drive system.

## Mechanical model of the scraper conveyor chain drive system

### Torsional pendulum dynamics model

**Figure 1:**
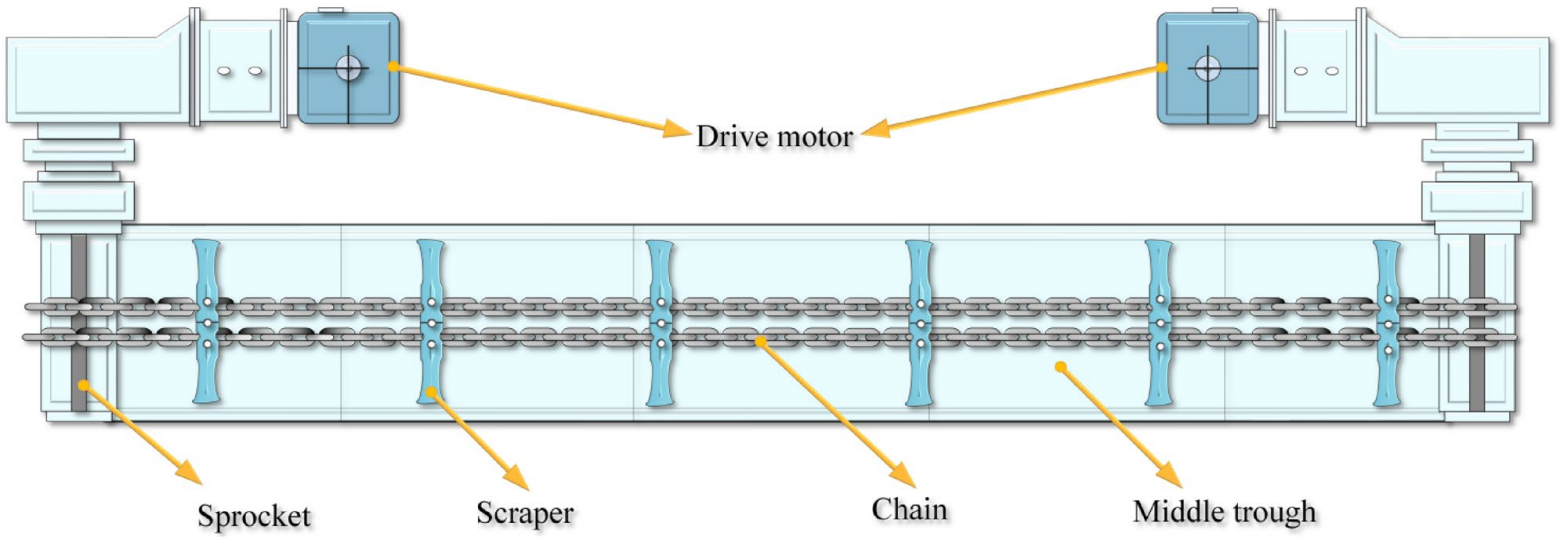
Mechanical model of the vibration of a torsion pendulum and a scraper force diagram.

Scraper conveyors are the main components of fully mechanized mining equipment.A scraper conveyor is a complex and highly coupled multi-body dynamics system. The working principle is to use the middle trough and the chain drive system to transport the coal. A drive motor propels the sprocket to rotate. The chain is engaged with the sprocket. The scraper is fixed on a chain as its traction component. As shown in Fig. 1, the scraper conveyor is mainly composed of the drive motor, middle trough, sprocket, scraper and chain.

**Figure 2:**
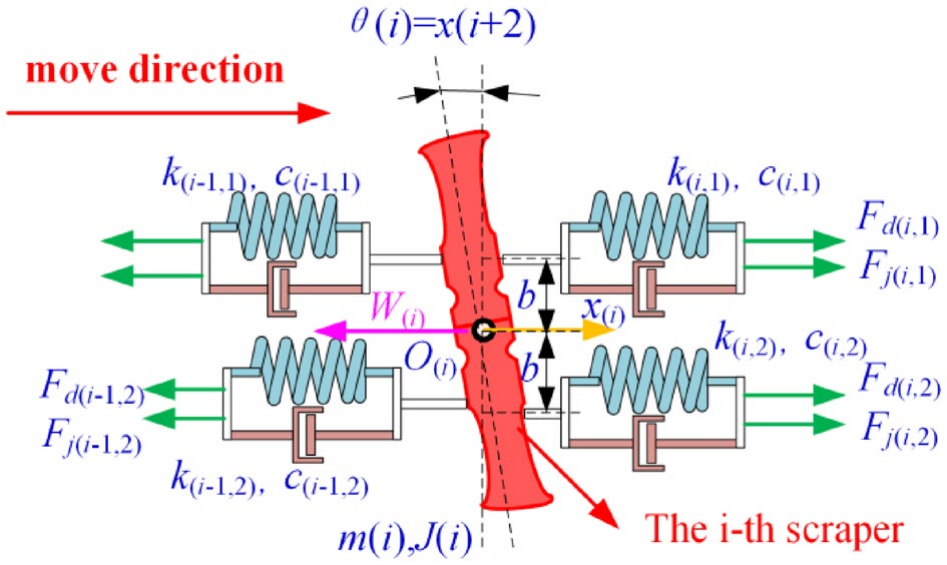
Mechanical model of the torsion pendulum vibration and a scraper force diagram.

The Kelvin‒Voigt model and the point-by-point tension method are used to establish a model and analyse the torsional vibration of the scraper chain drive system. The mass of the chain is distributed to the scraper. The mechanical model of the torsional pendulum vibration of the scraper and chain drive system and the force of the scraper are shown in Fig. 2, and its variable annotated table is shown in Table 1.

**Table 1:** Description of symbols in Fig. 2.

Symbol	Explanation
*i*	The i-th scraper
$\mathop {{m}}\nolimits_{{{i}}}$	The mass of the i-th discrete unit
*x*(*i*)	The translational displacement of the i-th scraper, (m)
*x*(*i* + 1)	The translational speed of the i-th scraper, (m/s)
*x*(*i* + 2)	The rotational angle *θ*(*i*), (rad)
*x*(*i* + 3)	The angular velocity of the i-th scraper, (rad/s)
*F*_*j*_(*i,*1)	The static tension of the first chain (near the coal wall side chain) behind the i-th scraper
*F*_*j*_(*i,*2)	The static tension of the second chain (the chain away from the coal wall side) behind the i-th scraper
*W*(*i*)	The resultant force on the i-th scraper
*F*_*d*_(*i,*1)	The dynamic tension of the first chain (near the coal wall side chain) behind the i-th scraper
*F*_*d*_(*i,*2)	The dynamic tension of the second chain (the chain away from the coal wall side) behind the i*-*th scraper
*c*(*i*,1)	The damping coefficient of the first chain (near the coal wall side chain) behind the i-th scraper
*c*(*i*,2)	The damping coefficient of the second chain (the chain away from the coal wall side) behind the i-th scraper
*k*(i,1)	The stiffness coefficient of the first chain (near the coal wall side chain); of the chain ring behind the i-th scraper
*k*(i,2)	The stiffness coefficient of the second chain (the chain away from the coal wall side) behind the i-th scraper
*θ*(*i*)	The angle of rotation of the scraper

The differential equation of motion is established for the i-th scraper:

1 $$\left\{ \begin{array}{*{20}l} m_{i} \ddot x_{i} = F_{i + 1,1} \frac{{{\text{sgn}} \left( {F_{i + 1,1} + f_{i + 1} } \right) + 1}}{2} + F_{i + 1,2} \frac{{{\text{sgn}} \left( {F_{i + 1,2} + f_{i + 1} } \right) + 1}}{2} - \hfill \\ \, F_{i,1} \frac{{{\text{sgn}} \left( {F_{i,1} + f_{i} } \right) + 1}}{2} - F_{i,2} \frac{{{\text{sgn}} \left( {F_{i,2} + f_{i} } \right) + 1}}{2} - F_{zi} \hfill \\ J_{i} \ddot{\theta }_{i} = \left[ {F_{i + 1,1} \frac{{{\text{sgn}} \left( {F_{i + 1,1} + f_{i + 1} } \right) + 1}}{2} - F_{i + 1,2} \frac{{{\text{sgn}} \left( {F_{i + 1,2} + f_{i + 1} } \right) + 1}}{2} - } \right. \hfill \\ \, F_{i,1} \frac{{{\text{sgn}} \left( {F_{i,1} + f_{i} } \right) + 1}}{2} + \left. {F_{i,2} \frac{{{\text{sgn}} \left( {F_{i,2} + f_{i} } \right) + 1}}{2} - F_{zi} } \right]\frac{L}{2}\cos \theta_{i} - F_{Yi} \frac{L}{2}\cos \theta_{i} \hfill \\ \end{array} \right.,$$

where

$$\begin{aligned} F_{i + 1,1} &= k_{i + 1,1} \left( {x_{i + 1} + \frac{L}{2}\sin \theta_{i + 1} - x_{i} - \frac{L}{2}\sin \theta_{i} } \right) + c_{i + 1,1} \left( {\dot x_{{_{i + 1} }} - \dot x_{{_{i} }} + \frac{L}{2}\dot \theta_{{_{i + 1} }} - \frac{L}{2}\dot \theta _{{_{i} }} } \right); \hfill \\ F_{i,1} &= k_{i,1} \left( {x_{i} + \frac{L}{2}\sin \theta_{i} - x_{i - 1} - \frac{L}{2}\sin \theta_{i - 1} } \right) + c_{i,1} \left( {\dot x_{{_{i} }} - \dot x_{{_{i - 1} }} + \frac{L}{2}\dot \theta _{{_{i} }} - \frac{L}{2}\dot \theta _{{_{i - 1} }} } \right); \hfill \\ F_{i,2} &= k_{i,2} \left( {x_{i} - \frac{L}{2}\sin \theta_{i} - x_{i - 1} + \frac{L}{2}\sin \theta_{i - 1} } \right) + c_{i,2} \left( {\dot x_{{_{i} }} - \dot x_{{_{i - 1} }} - \frac{L}{2}\dot \theta _{{_{i} }} + \frac{L}{2}\dot \theta _{{_{i - 1} }} } \right); \hfill \\ \end{aligned}$$

### Construction of the simulation model

The dynamic equation and state equation are established. That is, the function subroutine is constructed for the above established dynamic equation to realize the pre-processing of dynamic analysis. The translational coordinate *x*(*i*) and the rotational coordinate *θ*(*i*) are expressed by *x*(*i*). The generalized coordinate *x*(*i*) is convenient for solving the equation. The state function of the above dynamic equation is:

2 $$\left\{ \begin{array}{*{20}l} \dot x_{{_{\left( i \right)} }} = x_{{\left( {i + 1} \right)}} \hfill \\ \dot x_{{_{{\left( {i + 1} \right)}} }} = \left[ {F_{{\left( {i,1} \right)}} + F_{{\left( {i,2} \right)}} - F_{{\left( {i - 4,1} \right)}} - F_{{\left( {i - 4,2} \right)}} + W_{\left( i \right)} } \right]/m_{\left( i \right)} \hfill \\ \dot x_{{_{{\left( {i + 2} \right)}} }} = x_{{\left( {i + 3} \right)}} \hfill \\ \dot x_{{_{{\left( {i + 3} \right)}} }} = \left\{ {\left[ {\left. {F_{{\left( {i,1} \right)}} - F_{{\left( {i - 4,1} \right)}} + \left( {F_{{\left( {i - 4,2} \right)}} - F_{{\left( {i,2} \right)}} } \right)} \right]h + FM_{\left( i \right)} } \right]} \right\}/j_{\left( i \right)} \hfill \\ \end{array} \right.,$$

where

$$\begin{aligned} W_{\left( i \right)} = & m_{i} gf{\text{sgn}} (v_{0} + \dot{x}_{i} ) + m_{i} gf{\text{sgn}} \left( {v_{0} + \dot{x}_{i} + \dot{\theta }_{i} \frac{L}{2}} \right) \\ & { + }m_{i} gf{\text{sgn}} \left( {v_{0} + \dot{x}_{i} - \dot{\theta }_{i} \frac{L}{2}} \right) + m_{z} gf, \\ \end{aligned}$$

where *f* is the friction coefficient between the scraper and the middle trough; *h* is the distance from the tension position to the centre of the scraper; and *v*_*0*_ is the initial speed during stable operation. For tension *F*(*i*, 1) and *F*(*i*, 2) in Eq. (2), they are the sum of their static tension and dynamic tension. *F*(*i*, 1) = *F*_*d*_(*i*, 1) + *F*_*j*_(*i*, 1), *F*(*i*, 2) = *F*_*d*_(*i*, 2) + F_*j*_(*i*, 2). In general, *F*_*j*_(*i*, 1) and *F*_*j*_(*i*, 2) are fixed values. If the chain is not pre-tensioned before starting, then *F*(*i*, 1) = *F*_*d*_(*i*, 1), *F*(*i*, 2) = *F*_*d*_(*i*, 2). The mechanical models of dynamic tensions *F*_*d*_(*i*, 1) and *F*_*d*_(*i*, 2) have been described in Figur 2. The Eqs. (3), (4), (5) and (6) are the calculation equations of dynamic tension *F*_*d*_(*i*, 1) and *F*_*d*_(*i*, 2) respectively.

3 $$F_{d(i,1)} = k_{(i,1)} \{ x_{(i + 4)} + x_{(i + 6)} h - x_{(i)} - x_{(i + 2)} h\} ,$$

4 $$F_{d(i,2)} = k_{(i,2)} \{ x_{(i + 4)} + x_{(i + 6)} h - x_{(i)} - x_{(i + 2)} h\} .$$

When considering damping, the calculation of $F_{cd(i,1)}$ and $F_{cd(i,2)}$ is:

5 $$F_{cd(i,1)} = c_{(i,1)} \{ x_{(i + 5)} + x_{(i + 7)} h - x_{(i + 1)} - x_{(i + 3)} h\} ,$$

6 $$F_{cd(i,2)} = c_{(i,2)} \{ x_{(i + 5)} + x_{(i + 7)} h - x_{(i + 1)} - x_{(i + 3)} h\} .$$

To prevent the chain from being compressed, the following condition is necessary:

7 $$k_{(i,j)} = 0.5\left\{ {sign\left[ {F_{d(i,j)} + F_{j(i,j)} } \right] + 1} \right\}k_{(i,j)} \;{\kern 1pt} (j = 1,2;i = 1 \ldots 4n - 3).$$

That is, when the chain tension is negative, the stiffness of the chain is zero.

### Longitudinal-torsional coupling

To establish a dynamic model of the coupled longitudinal-torsional modes of the scraper conveyor, the following assumptions are necessary:

1. The influence of the dynamic tension of the front and rear sprockets is ignored.
2. The mass of each scraper is the sum of the mass of the scraper and the chain of the connecting section and evenly distributed on each branch.
3. The rotational inertia of the scraper is the sum of the automatic rotational inertia of the scraper and the rotational inertia of the chain.

**Figure 3:**
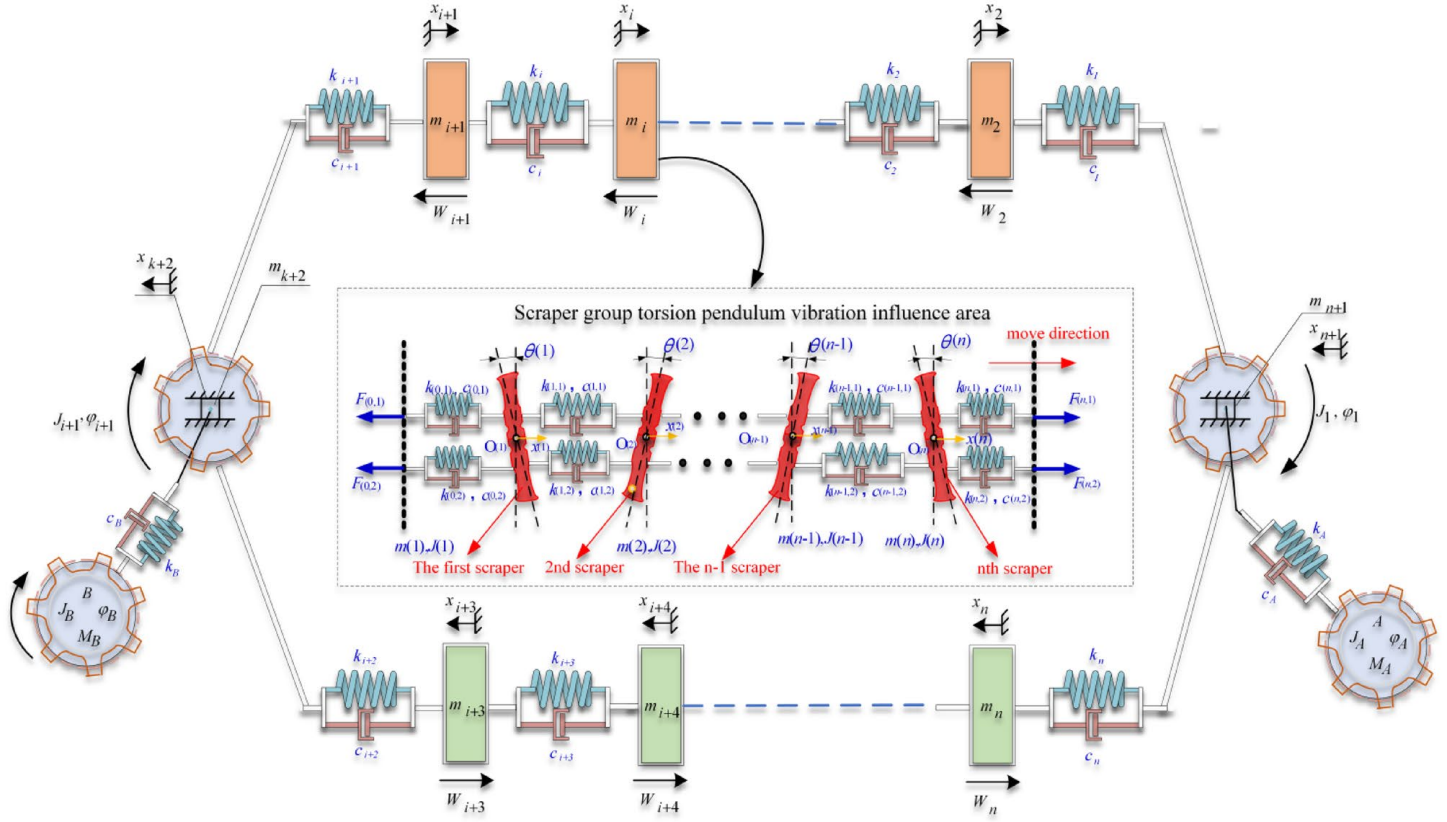
Discrete dynamic model of the coupled longitudinal torsional modes of the scraper chain drive system.

Using the finite element method, the double-chain transmission system of the scraper group is divided into several segments. The Kelvin–Voigt model is used to connect the segments and then connect them with the head–tail double-end drive system to construct a discrete dynamic model of the coupled longitudinal-torsional modes of the scraper chain drive system, as shown in Fig. 3. Its variable annotated table is shown in Table 2.

**Table 2:** Description of symbols in Fig. 3.

Symbol	Explanation
$\mathop J\nolimits_{A} ,\mathop J\nolimits_{B}$	The rotational inertia of the head drive motor and the tail drive motor
$\mathop \varphi \nolimits_{A} ,\mathop \varphi \nolimits_{B}$	The rotation angle of the head drive motor and the tail drive motor
$\mathop M\nolimits_{A} ,\mathop M\nolimits_{B}$	The torque of the head drive motor and the tail drive motor
$\mathop c\nolimits_{A} ,\mathop c\nolimits_{B}$	Equivalent damping coefficient of the head drive motor and the tail drive motor
$\mathop k\nolimits_{A} ,\mathop k\nolimits_{B}$	Equivalent stiffness coefficient of the head drive motor and the tail drive motor
$\mathop R\nolimits_{A} ,\mathop R\nolimits_{B}$	Pitch radius of the head drive motor and the tail drive motor
$\mathop {{m}}\nolimits_{{{i}}}$	The mass of the i-th discrete unit

According to the discrete dynamics model of the coupled longitudinal-torsional modes of the scraper chain drive system shown in Fig. 3, the following Eq. (8) is established.

8 $$\left. \begin{array}{*{20}c} J_{A} \ddot{\varphi }_{A} + k_{A} (\varphi_{A} - \varphi_{1} ) + c_{A} (\dot{\varphi }_{A} - \dot{\varphi }_{1} ) = M_{A} \hfill \\ J_{1} \ddot{\varphi }_{1} + k_{A} (\varphi_{1} - \varphi_{A} ) + c_{A} (\dot{\varphi }_{1} - \dot{\varphi }_{A} ) + (F_{1(t)} - F_{n(t)} )R_{A} = 0 \hfill \\ \frac{{d(m_{2} \dot{x}_{2} )}}{dt} - F_{1} (t) + F_{2} (t) + W_{2} = 0 \hfill \\ \vdots \hfill \\ J_{B} \ddot{\varphi }_{B} + k_{B} (\varphi_{B} - \varphi_{i + 2} ) + c_{B} (\dot{\varphi }_{B} - \dot{\varphi }_{i + 2} ) = M_{B} \hfill \\ J_{n} \ddot{\varphi }_{n} + k_{B} (\varphi_{i + 2} - \varphi_{B} ) + c_{B} (\dot{\varphi }_{i + 2} - \dot{\varphi }_{B} ) + (F_{i + 2} - F_{i + 1} )R_{n} = 0 \hfill \\ \frac{{d(m_{i + 3} \dot{x}_{i + 3} )}}{dt} + F_{i + 3(t)} - F_{i + 2(t)} - W_{i + 3} = 0 \hfill \\ \vdots \hfill \\ \frac{{d(m_{n} \dot{x}_{n} )}}{dt} + F_{n(t)} - F_{n - 1(t)} - W_{n} = 0 \hfill \\ \end{array} \right\},$$

where

$$F_{1(t)} = k_{1} (\varphi_{1} R_{1} - x_{2} - x_{n + 1} ) + c_{1} (\dot{\varphi }_{1} R_{1} - \dot{x}_{2} - \dot{x}_{n + 1} ),$$

$$F_{j(t)} = k_{j} (x_{j} - x_{j + 1} ) + c_{1} (\dot{x}_{j} - \dot{x}_{j + 1} )\begin{array}{*{20}c} {} & {i \ge j \ge 2} \\ \end{array} {\text{ or}} {\kern 1pt} {\kern 1pt} {\kern 1pt} n - 1{\kern 1pt} {\kern 1pt} {\kern 1pt} {\kern 1pt} {\kern 1pt} \ge j \ge i + 3,$$

$$F_{i + 1(t)} = k_{i + 1} (x_{i + 1} - \varphi_{i + 2} R_{i + 2} + x_{k + 2} ) + c_{i + 1} (\dot{x}_{i + 1} - \dot{\varphi }_{i + 2} R_{i + 2} + \dot{x}_{k + 2} ),$$

$$F_{i + 2(t)} = k_{i + 2} (\varphi_{i + 2} R_{i + 2} + x_{k + 2} - x_{i + 3} ) + c_{i + 2} (\dot{\varphi }_{i + 2} R_{i + 2} + \dot{x}_{k + 2} - \dot{x}_{i + 3} ),$$

$$F_{n(t)} = k_{n} (x_{n} - \varphi_{1} R_{1} + x_{n + 1} ) + c_{n} (\dot{x}_{n} - \dot{\varphi }_{1} R_{1} + \dot{x}_{n + 1} ).$$

## Attenuation of the torsional vibration stress wave

Failure conditions such as the material loading process and chain breaking cause fluctuations in the running speed of the scraper chain drive system, the torsional vibration of the scraper, and the chain tension before and after the scraper. In the process of torsional pendulum vibration of the scraper, if each scraper and its connecting chain are regarded as a unit, it will make the model difficult to solve. Therefore, determining the maximum number of scrapers of torsional pendulum vibration units in different sections along the conveyor is the key to realizing the numerical solution of the model.

### Attenuation of the stress wave under normal conditions

In its normal working condition, the scraper machine runs flat, and there is no failure, such as chain sticking and chain breaking. The main reason affecting the longitudinal-torsional vibration of the scraper chain drive system is excitation by the cargo load. In this section, the attenuation of the torsional vibration stress wave of the scraper chain drive system under a light load and a medium load are simulated for a changing cargo load.

Coal is cut by the spiral drum of the shearer and loaded onto the scraper conveyor. For the scraper chain drive system, this is equivalent to a sudden load^12^. Since the distribution of the coal in the direction of the broken chain of the scraper conveyor is initially uneven, this causes the offset load of the scraper and the chain.

**Figure 4:**
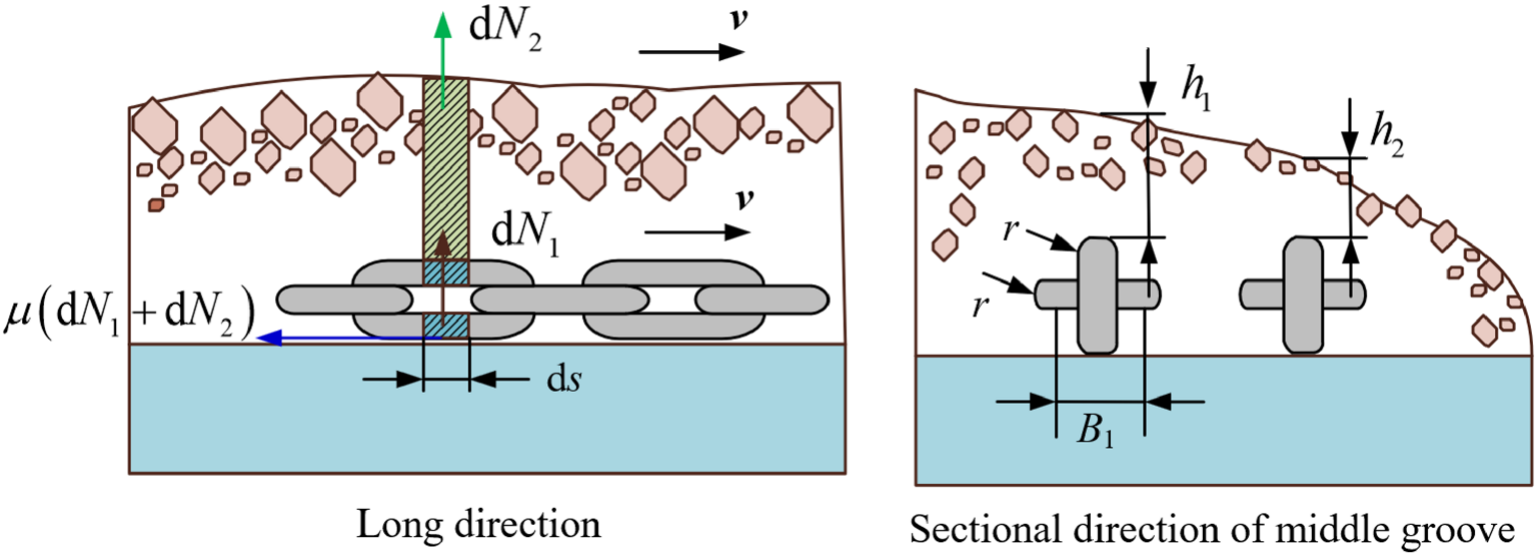
Sketch of the friction resistance of the running chain.

The number of scrapers in the scraper chain drive system is selected as *n* = 30. The simulation chain is broken at the 15th scraper, *i* = 15. The load on the scraper conveyor directly affects its running resistance. A sketch of the friction resistance of the running chain is shown in Fig. 4.

**Figure 5:**
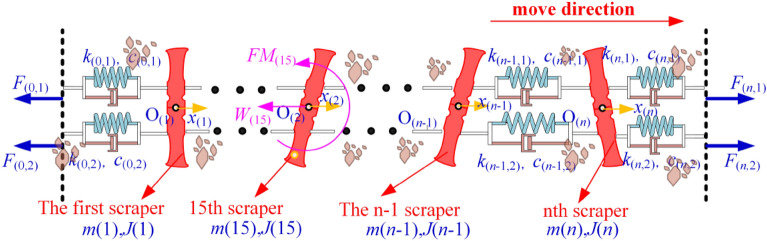
Light load condition.

**Figure 6:**
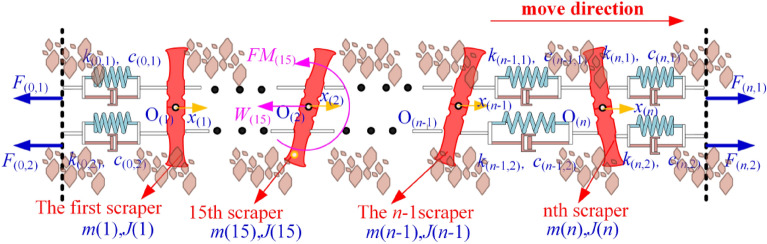
Medium load condition.

**Figure 7:**
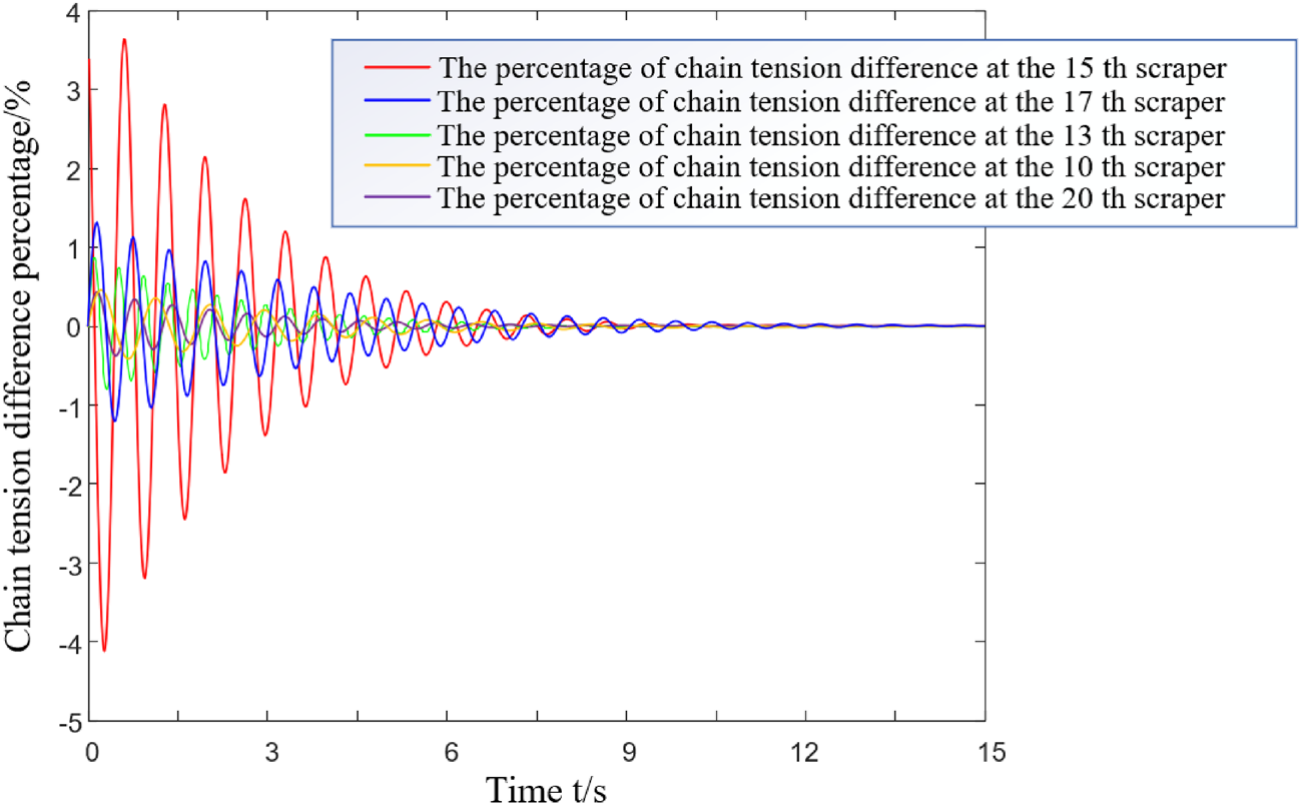
Vibrational response of a torsional pendulum of the scraper group under light load conditions.

**Figure 8:**
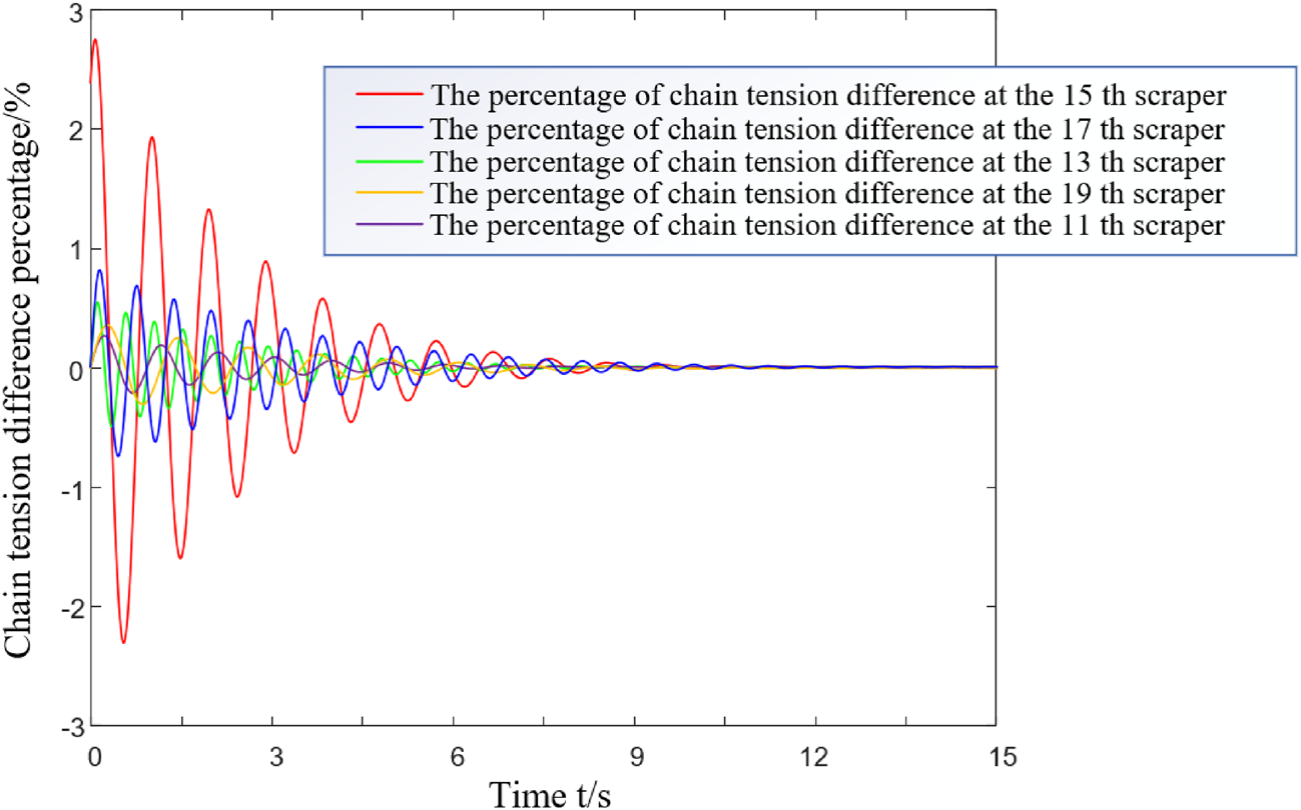
Vibrational response of a torsional pendulum of the scraper group under medium load conditions.

The formula for the numerical simulation of the running resistance of the unit section is as follows^22^:

9 $${\text{d}}F_{{\text{u}}} { = }\mu \left( {{\text{d}}N_{1} + {\text{d}}N_{2} } \right) = \mu \left( {\gamma_{{2}} {\text{g}} + \rho {\text{g}}\left( {\frac{{B_{1} + 4{\text{r}}}}{2}} \right)h_{{\text{u}}} } \right){\text{d}}s,\;{\text{u = 1}},{2,}$$

where *h*_*u*_ is the height of the coal bulk material directly above the chain, and *u* = 1 and 2 for the first chain and the second chain, respectively. *γ*_2_ is the mass per unit length of the scraper chain, kg/m. *B*_1_ is the chain centre distance, m. *r* is the diameter of the ring chain, m. *s* is the distance between two adjacent scrapers, m. The running resistance of a simplified unit section is applied as a step load to the 15th scraper of the model. In this paper, the SGZ1000/1050 scraper conveyor is taken as the research object. The main parameters of the dynamic characteristic simulation model are shown in Table 3. The analysis models of the light load and medium load conditions are shown in Figs. 5 and 6. The attenuation characteristics of the torsional vibration stress wave of the whole scraper chain drive system are numerically solved. The simulation results are shown in Figs. 7 and 8.

**Table 3:** Main parameters involved in the simulation.

Parameter	Symbol	Value	Unit
Height of upper trough in middle trough	*h*_*c*_	0.122	m
Quality of scraper chain per unit length	γ_2_	172	kg/m
Chain centre distance	*B*_1_	0.16	m
Distance from chain axis to scraper center	*b*	0.12	m
Ring chain diameter	R	0.038	m
Distance between two adjacent scrapers	*s*	1.008	m

As shown in Fig. 7, the relative difference between the 20th scraper in front of the excitation point and the 10th scraper in back is less than 0.5%. Thus, the area affected by torsional pendulum vibration under such conditions is considered to be [*i* − 5, *i* + 5]. As shown in Fig. 8, under medium load conditions, the attenuation of the evaluation index of the stress wave of the torsion pendulum for the 19th scraper in front of the excitation point and that of the 11th scraper behind it are less than 0.5%. Additionally, the influence area of torsion pendulum vibration under this condition is considered to be [*i* − 4, *i* + 4], where i represents the excitation applied at the i-th scraper. In subsequent research on the torsion pendulum vibration of the scraper conveyor, the scraper in the affected area can be considered as a whole. The remaining scraper is not affected by torsion pendulum vibration excitation and can be divided into several units on average to simplify the dynamic model of the machine. In conclusion, the torsion oscillation of the scraper chain drive system is more obvious under the light load than the medium load. That is, the influence area is larger, and the established equivalent model contains more scrapers.

### Coupled vibration analysis under cargo load excitation

**Figure 9:**
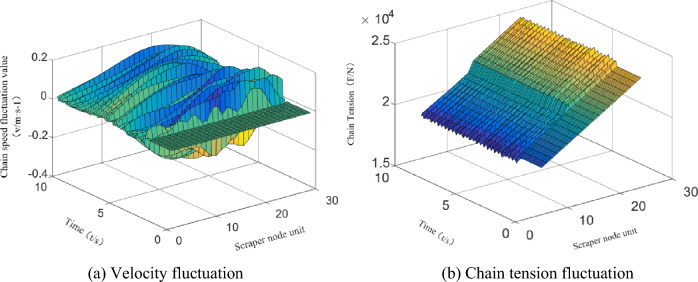
Velocity fluctuations and tension fluctuations of the unit section under cargo load excitation.

Through numerical simulation, the vibration velocity and tension fluctuation of each unit section of the scraper conveyor and the torsional vibration of each scraper in the unit section of the torsional vibration excitation are obtained. Load excitation is applied in the middle of the scraper conveyor, assuming that there is no material on the scraper conveyor before the load excitation. The speed fluctuations and tension fluctuations of each unit section are shown in Fig. 9.

**Figure 10:**
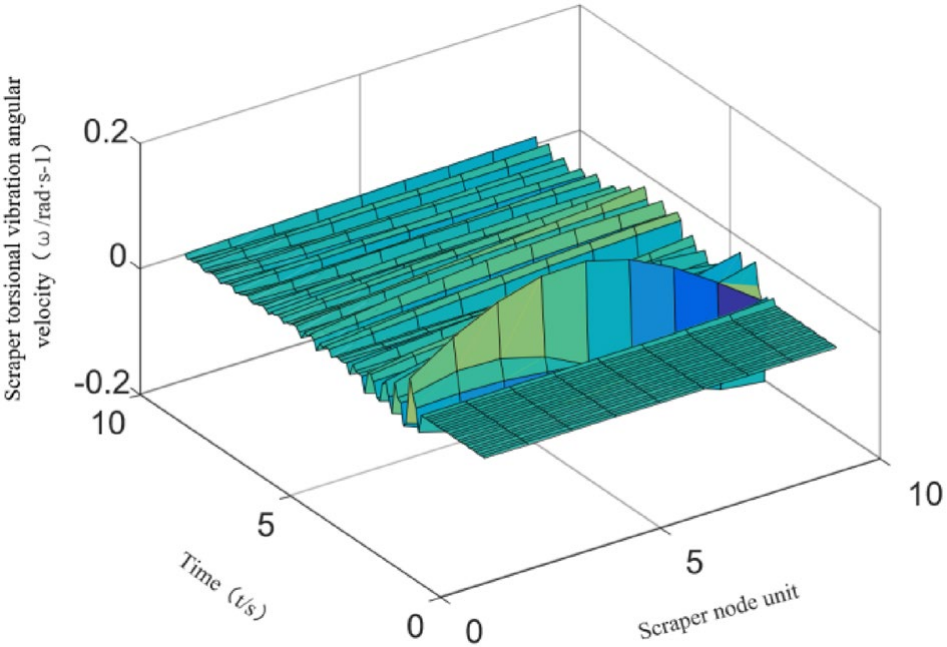
The angular velocity of the torsional vibration of the unit section under cargo load excitation.

In the section of the excited by the impact load, the torsional vibration angular velocity of each chain is shown in Fig. 10.

According to previous research, the five scrapers before and after the loading excitation are the area influenced by the vibration of the torsional pendulum, and thus there are 10 scrapers in the excited section. The simulation results show that cargo load excitation causes longitudinal vibration of the scraper conveyor, resulting in fluctuations in the running speed and chain tension. The most violent fluctuations of the scraper running speed and the chain tension occur where the cargo load excitation is applied, causing a maximum of 119.5% speed fluctuation and 78.6% tension fluctuation. The cargo load excitation in the section of the cargo load excitation unit causes torsional vibration of the scraper chain drive system, resulting in fluctuation of the tension difference between the two chains in the scraper chain drive system. The maximum percentage of tension difference between chain 1 and chain 2 is 8.6%. Further research shows that the material loading process causes slight torsional vibration of the scraper chain drive system, and the torsional vibration is more obvious when there is initially no material in the section of the scraper chain drive system.

## Torsional pendulum vibration experiment

### Experimental equipment and test device

**Figure 11:**
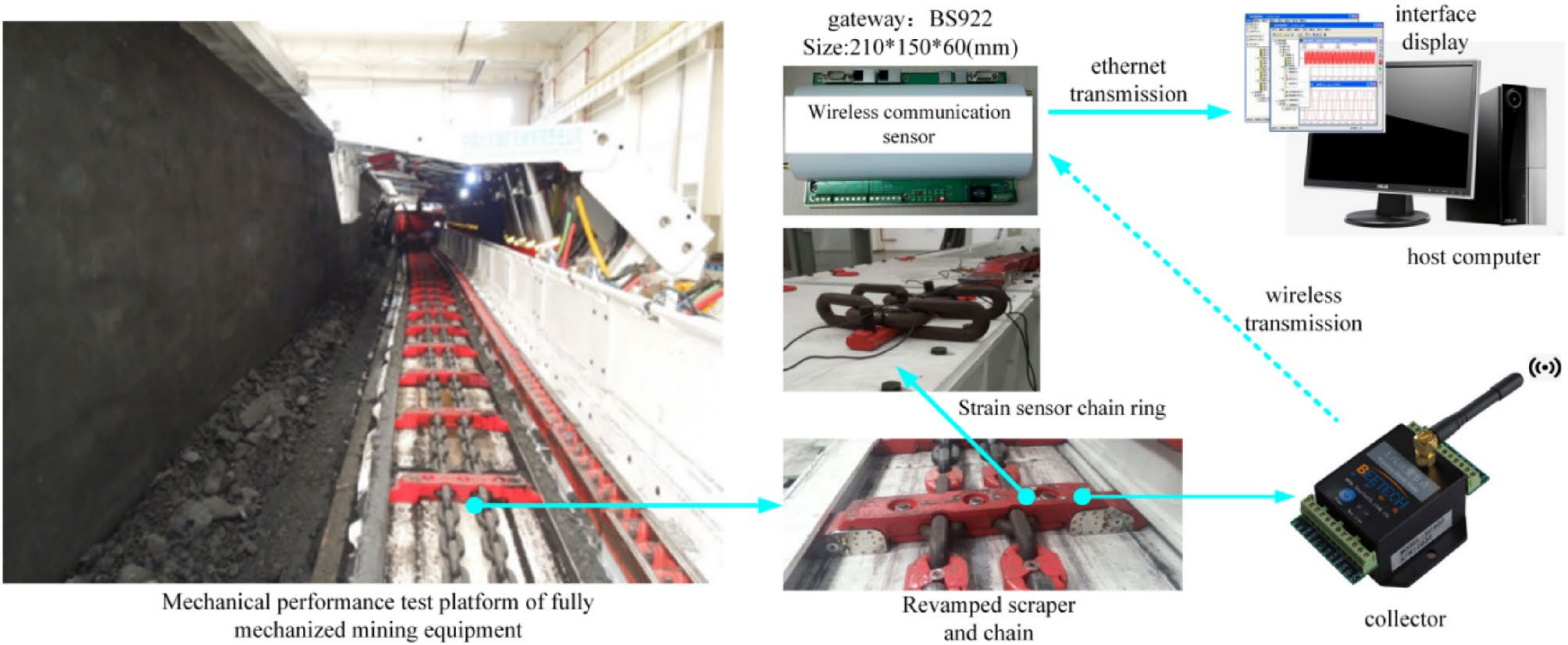
Overall structure diagram of the chain tension monitoring experimental system.

In this study, experiments are performed in the R & D (Experimental) Center of the National Energy Administration. This study relies on the simulated test platform of fully mechanized mining equipment that was jointly built by our school and China Coal Equipment Company. The torsional vibration characteristics of the scraper conveyor are experimentally studied. The overall structure diagram is shown in Fig. 11.

**Figure 12:**
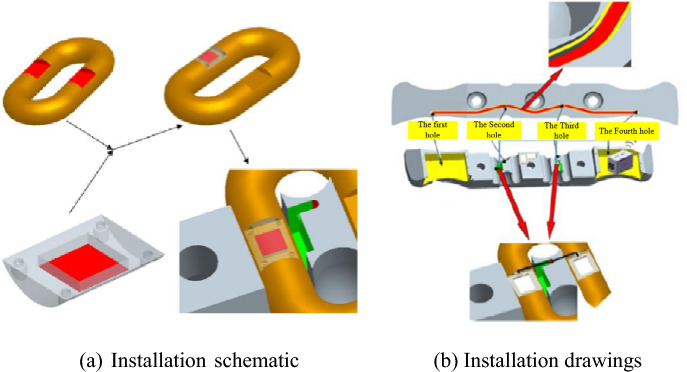
Schematic diagram of the installation of the strain gauge and ring to the data acquisition and transmission system.

To test the stress and strain of the chain in the torsional vibration process of the scraper chain drive system under different working conditions, a stress and strain test device for the chain ring is set up in the experimental platform. When the scraper conveyor operates, the chain force test mainly includes the dynamic tension fluctuation of the chain under different working conditions and the change in the tension value. For monitoring, the strain gauge is attached to the chain flat ring chain. Figure 12a shows the strain gauge installation method. To make the scraper and the chain ring mesh more accurately, the strain gauge is attached to the outer side of the milling flat chain ring, and protection is needed. The wireless data acquisition module is placed in the scraper and connected with the strain gauge^22^. The changing value of the strain gauge is collected in real time, as shown in Fig. 12b.

### Analysis of test results

The chain tension and scraper vibration during the operation of the scraper conveyor are measured by using the experimental platform. In this paper, only the chain tension of the scraper conveyor under normal material carrying conditions is tested and analysed.

Before the test, the data measured by each sensor should be calibrated, and the load calculation formula is obtained by fitting the strain value. The data calibration is conducted for the chain ring tension sensors numbered C1 and C2. Rated loads of 400 kN, 600 kN and 800 kN are applied to the chain ring by using the loading equipment. The measured microstrain data are shown in Table 4.

**Table 4:** Chain tension sensor data calibration.

Sensor number	Load value (kN)	Microstrain (με)
C1	400	2427
	600	3471
	800	5066
C2	400	− 2759
	600	− 3939
	800	− 5641

By fitting the load value and microstrain data, the calibration of the sensor is completed, and the relationship between the chain force and the microstrain measured by the sensor is obtained,

10 $$F_{C1} = - 1.781e - 5 \times {\text{CH}}_{C1}^{2} { + }0.3167 \times {\text{CH}}_{C1} - 598.8,$$

where CH_C1_ is the microstrain measured by the No. C1 sensor, and F_C1_ is the load value corresponding to the strain, kN.

11 $$F_{C2} = - 1.804e - 5 \times {\text{CH}}_{C2}^{2} - 0.2903 \times {\text{CH}}_{C2} - 263.6,$$

where CH_C2_ is the microstrain measured by the No. C1 sensor, and F_C1_ is the load value corresponding to the strain, kN.

**Figure 13:**
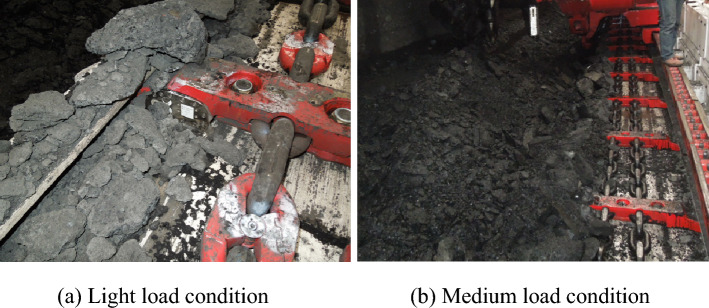
On-site test of the light and medium load conditions.

The calibrated chain tension sensor was used to test the chain tension under light load and medium load conditions. The test site of light load and medium load conditions is shown in Fig. 13.

**Figure 14:**
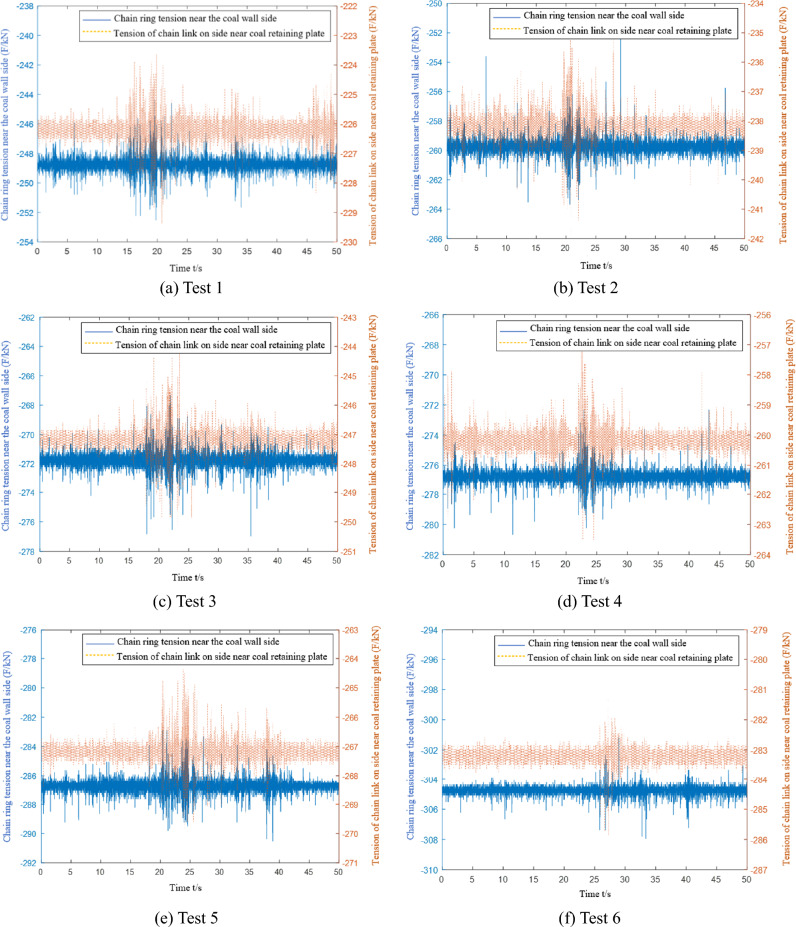

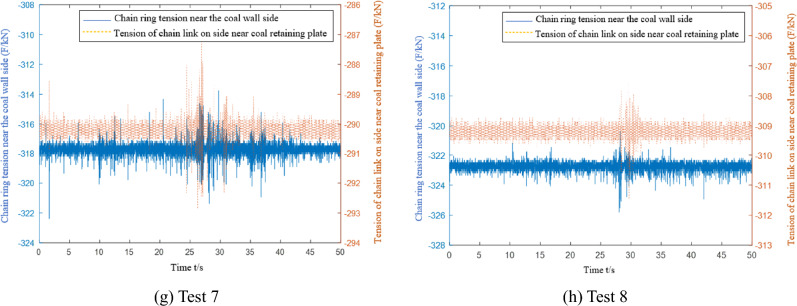
Data acquisition of the chain tension sensor under light load conditions.

When conducting multiple light load tests, the microstrain and converted tension curves of the C1 and C2 chain tension sensors are collected, as shown in Fig. 14. The C1 sensor is installed in the chain ring near the side of the coal wall, and the C2 sensor is installed in the chain ring near the side of the coal baffle.

**Figure 15:**
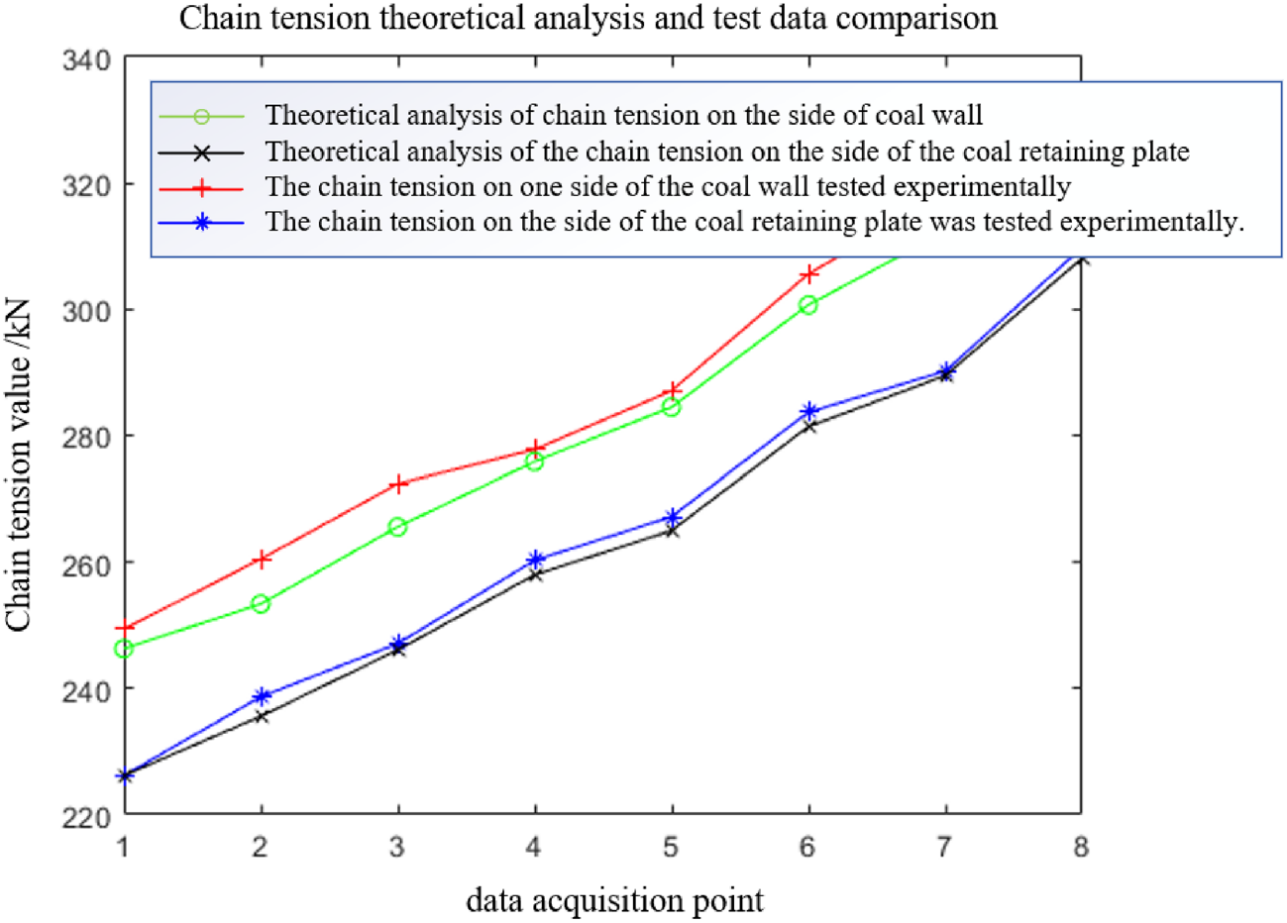
Comparison of theoretical and experimental chain tensions under light load conditions.

The data from eight tests are collected when the scraper of the sensor is running to different positions. The first test data acquisition point is closest to the tail, and the eighth test data acquisition point is closest to the nose. Table 5 shows that the chain tension measured by the sensor in the eight tests gradually increases. The experimental data are compared with the theoretical analysis data in Fig. 15.

**Table 5:** Average value of chain ring tension data under light load and medium load conditions.

Test point number	Light load condition (kN)		Medium load condition (kN)	
	Near coal wall side	Close to the side of the coal baffle	Near coal wall side	Close to the side of the coal baffle
1	249.4	226.2	288.3	265.2
2	260.5	238.7	296.4	271.6
3	272.4	247.1	304.6	277.1
4	277.8	260.3	318.2	290.5
5	287.1	267.2	330.6	307.7
6	305.6	283.7	334.5	305.4
7	318.2	290.2	352.1	320.5
8	323.6	309.8	358.2	333.1

During each data acquisition, the average value of the difference between the side chain ring near the coal wall and the side chain ring near the coal baffle is 21.4 kN, and the extreme value is 27.9 kN. The average value of the tension difference between the side chain ring near the coal wall and the side chain ring near the coal baffle is 19.2 kN, and the extreme value is 23.2 kN. There are 11.3% and 16.8% errors between the experimental and the theoretical results. The tension of the chain undergoes many severe tension fluctuations during the data acquisition period. The main reason is that the scraper encounters large materials or collides with the middle trough during operation, which increases the unilateral load of the scraper and causes the scraper to undergo torsional vibration.

**Figure 16:**
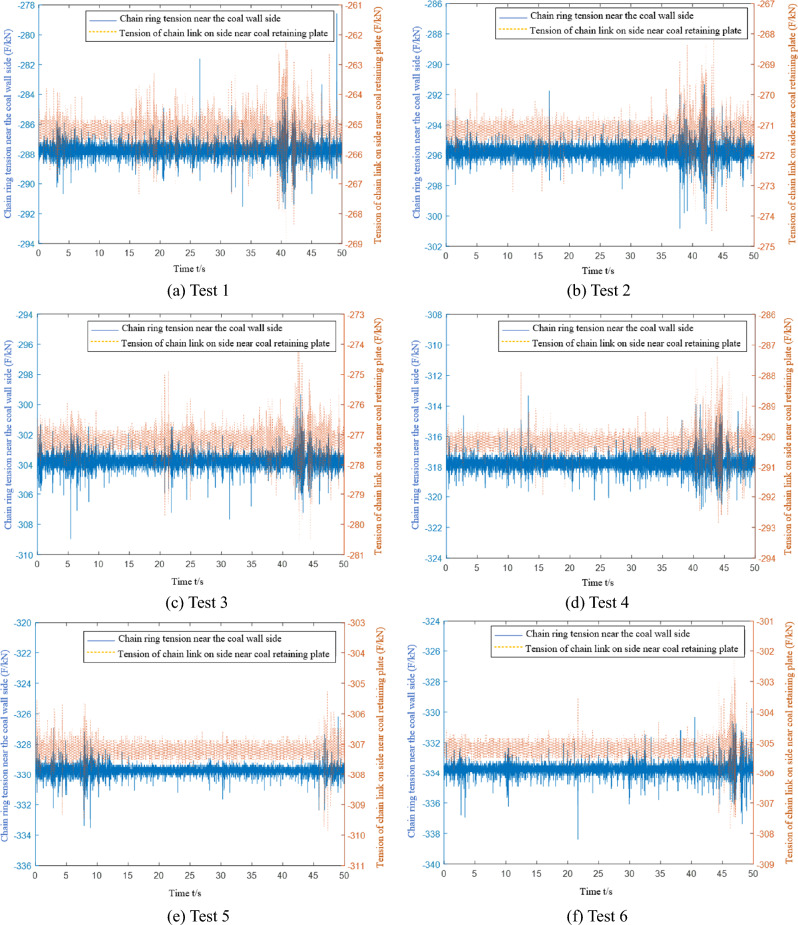

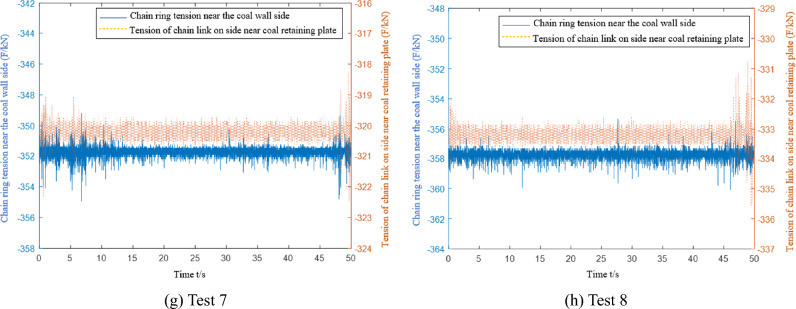
Data acquisition of the chain tension sensor under medium load conditions.

The microstrain and converted tension curves of the C1 and C2 chain-ring tension sensors are collected during multiple mid-load condition tests, as shown in Fig. 16.

**Figure 17:**
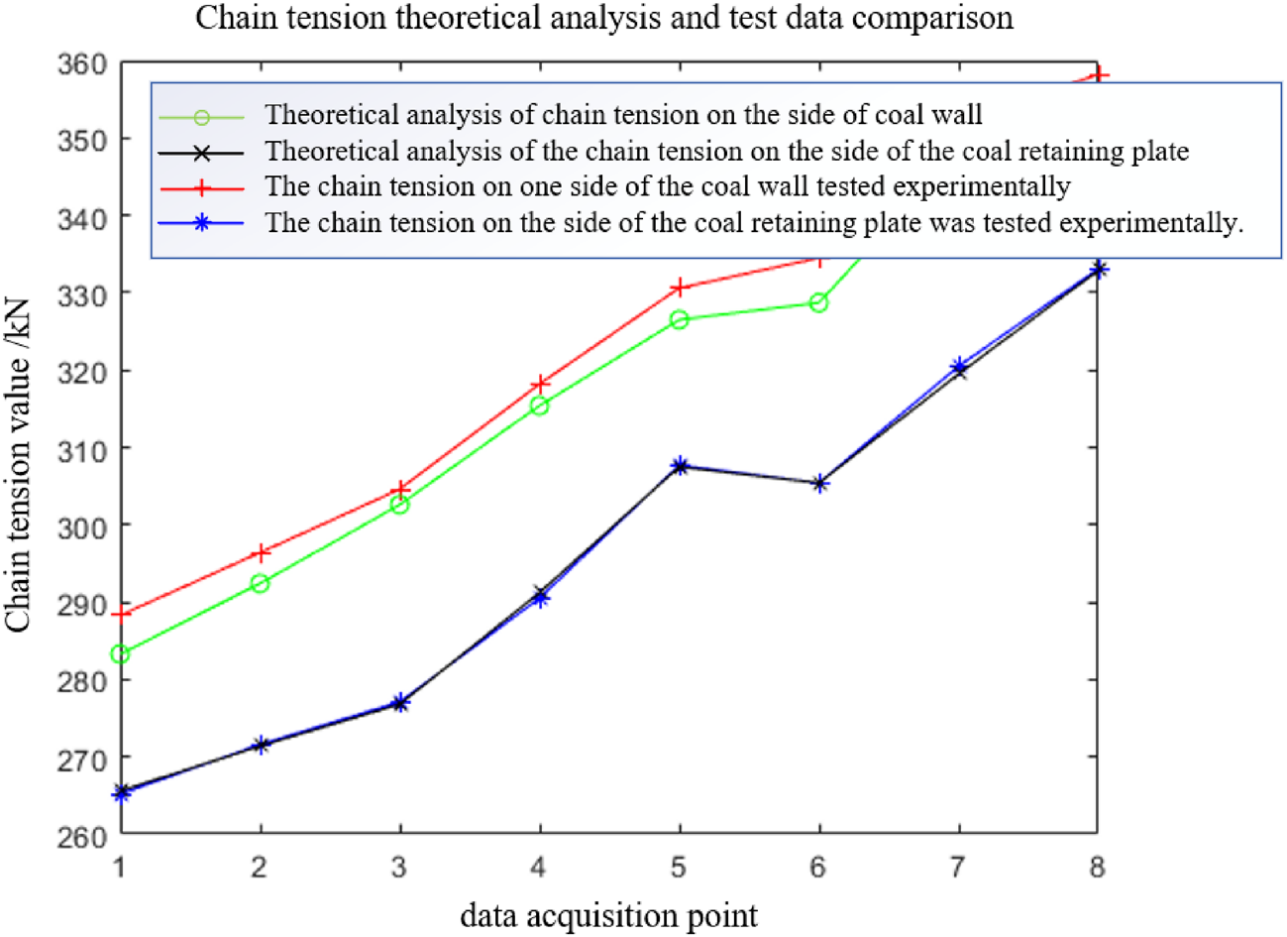
Under the condition of medium load, the theoretical analysis of chain tension is compared with the test data.

The data from the eight tests under the medium load condition are similar to the eight test data under the light load condition. Data are collected when the scraper installed with the sensor runs to different positions. The first test data acquisition point is closest to the tail of the machine, and the eighth test data acquisition point is closest to the head. Table 5 shows that the chain tension measured by the sensor in the eight test data gradually increases. Because more coal material is pushed by the scraper under the medium load than the light load condition, the chain tension value detected by the chain tension sensor on both sides of the scraper is higher than that under the light load condition. The theoretical analysis data are compared with the experimental data in Fig. 17.

During each data acquisition, the average value of the difference between the side chain ring near the coal wall and the side chain ring near the coal baffle is 26.5 kN, and the extreme value is 32.3 kN. The average value of the tension difference between the side chain ring near the coal wall and the side chain ring near the coal baffle is 23.1 kN, and the extreme value is 29.4 kN. There are 13.8% and 10.3% errors between the experimental results and the theoretical analysis results. Additionally, the chain tension test data show that the tension of the chain undergoes many severe fluctuations during the data acquisition period. The main reason is that the scraper encounters large materials or collides with the middle trough during operation, which increases the unilateral load of the scraper and causes it to undergo torsional vibration.

Due to the limitations of various factors, experimental research does not fully correspond to the various working conditions in theoretical research. There are also some errors between the experimental and the theoretical results, but the two have the same trend. The torsional vibration characteristics of the scraper chain drive system of the scraper conveyor under various working conditions and the influence of the torsional pendulum vibration on the load of the chain ring can be analysed by theory and simulation, which provides the basis for the subsequent prediction of chain life and calculation of safety factors.

## Conclusion

In this study, the Kelvin–Voigt model and the point-by-point tension method were used to establish a mechanical model of the coupled longitudinal and torsional vibrations of the scraper chain drive system. The longitudinal and torsional vibration characteristics of the scraper chain drive system under different load excitations were studied by numerical simulation combined with experimental verification in the field. Under light load conditions, the influence area of torsional vibration was from Section 5 in front of the excitation point to Section 5 behind the excitation point. Under the condition of medium load, the influence area of torsional vibration was from Section 4 in front of the excitation point to Section 4 behind the excitation point.

The research results showed that excitation by the cargo load caused longitudinal vibration of the scraper conveyor, resulting in fluctuations in the running speed and chain tension. The most violent fluctuations of the scraper running speed and the chain tension were the result of the application of the cargo load excitation, which caused a maximum speed fluctuation of 119.5% and a maximum tension fluctuation of 78.6%. Excitation of a section by the cargo caused torsional vibration of that section of the scraper chain drive system, resulting in fluctuations in the tension difference between the two chains in the scraper chain drive system. The maximum percentage of tension difference between chain 1 and chain 2 was 8.6%. Further research showed that the cargo loading process caused slight torsional vibration of the scraper chain drive system, and the torsional vibration was more obvious when there was initially no cargo.

## Data Availability

All data generated or analysed during this study are included in this published article [and its Supplementary Information files].
